# Impact of Chronic Neonicotinoid Exposure on Honeybee Colony Performance and Queen Supersedure

**DOI:** 10.1371/journal.pone.0103592

**Published:** 2014-08-01

**Authors:** Christoph Sandrock, Matteo Tanadini, Lorenzo G. Tanadini, Aline Fauser-Misslin, Simon G. Potts, Peter Neumann

**Affiliations:** 1 Agroscope, Swiss Bee Research Centre, Berne, Switzerland; 2 Seminar for Statistics, ETH Zurich, Zurich, Switzerland; 3 Division of Biostatistics, Institute for Social and Preventive Medicine, University of Zurich, Zurich, Switzerland; 4 Institute of Bee Health, Vetsuisse Faculty, University of Berne, Berne, Switzerland; 5 School of Agriculture, Policy and Development, University of Reading, Reading, United Kingdom; French National Institute for Agricultural Research (INRA), France

## Abstract

**Background:**

Honeybees provide economically and ecologically vital pollination services to crops and wild plants. During the last decade elevated colony losses have been documented in Europe and North America. Despite growing consensus on the involvement of multiple causal factors, the underlying interactions impacting on honeybee health and colony failure are not fully resolved. Parasites and pathogens are among the main candidates, but sublethal exposure to widespread agricultural pesticides may also affect bees.

**Methodology/Principal Findings:**

To investigate effects of sublethal dietary neonicotinoid exposure on honeybee colony performance, a fully crossed experimental design was implemented using 24 colonies, including sister-queens from two different strains, and experimental in-hive pollen feeding with or without environmentally relevant concentrations of thiamethoxam and clothianidin. Honeybee colonies chronically exposed to both neonicotinoids over two brood cycles exhibited decreased performance in the short-term resulting in declining numbers of adult bees (−28%) and brood (−13%), as well as a reduction in honey production (−29%) and pollen collections (−19%), but colonies recovered in the medium-term and overwintered successfully. However, significantly decelerated growth of neonicotinoid-exposed colonies during the following spring was associated with queen failure, revealing previously undocumented long-term impacts of neonicotinoids: queen supersedure was observed for 60% of the neonicotinoid-exposed colonies within a one year period, but not for control colonies. Linked to this, neonicotinoid exposure was significantly associated with a reduced propensity to swarm during the next spring. Both short-term and long-term effects of neonicotinoids on colony performance were significantly influenced by the honeybees’ genetic background.

**Conclusions/Significance:**

Sublethal neonicotinoid exposure did not provoke increased winter losses. Yet, significant detrimental short and long-term impacts on colony performance and queen fate suggest that neonicotinoids may contribute to colony weakening in a complex manner. Further, we highlight the importance of the genetic basis of neonicotinoid susceptibility in honeybees which can vary substantially.

## Introduction

Ecosystem services provided by pollinating insects are vital for the maintenance of biodiversity [1], [2] and food security through agricultural productivity [3], [4], [5], [6]. Recent evidence for globally paralleling declines of various pollinators [7], [8], [9], [10], however, stands in contrast to prospects of continuously increasing demands for pollination services [4], [11], [12].

The Western honeybee, *Apis mellifera*, is the predominant managed pollinator worldwide [4], [8]. Although global stocks of domestic honeybees have increased during the last half century (except in Europe [13] and in the USA [14]), present and predicted agricultural demands for insect pollination far exceed currently available capacities [15]. Repeated, massive declines of managed honeybee colonies during the last decade in North America, Europe and the middle East [8], [16], [17], [18], [19], [20], [21], [22] raised substantial concerns about safeguarding future honeybee pollination services. Despite comprehensive recent research, conclusive evidence for common causal drivers is lacking, which points at multiple interacting factors [8], [23], [24]. The invasive ectoparasitic mite *Varroa destructor* represents a severe problem for honeybees almost worldwide [25], [26], [27], in particular due to its vital role as a virus vector [28], [29]. Some observations of elevated colony losses were also influenced by the widespread gut parasites *Nosema ceranae* [30], [31], [32]. Microbes and *V. destructor*-associated viruses are prevalent almost globally and commonly co-occurring, yet there is no uniform evidence so far that even the interactions between some of these pathogenic stressors necessarily result in colony failure [33], [34], [35], [36]. Instead, it appears as if more complex interactions with additional environmental stressors and at multiple levels are of key importance [8], [24]. Likely candidates, commonly encountered by honeybees, are routinely applied agricultural pesticides [37], [38]. For instance, the use of systemic neonicotinoid insecticides has strongly increased on a global scale during the last decade [39], [40], [41], [42]. Mainly acting as specific agonists of the insect acetylcholine receptors, neonicotinoids disrupt neuromuscular signalling pathways and are thus efficiently used for controlling insect pests [39], [43]. Systemic compounds like neonicotinoids can be particularly problematic for pollinating insects through exposure to residues in nectar and pollen of treated crops [41], [44], [45]. Although field-realistic neonicotinoid residue levels in pollen and nectar are generally assumed to result in sublethal dietary exposure [46], sublethal effects on honeybees include various negative impacts, such as impairment of physiology, cognitive abilities like memory and learning, and foraging and homing behaviour [45], [47], [48], [49], [50], [51], [52], [53], [54], [55], [56], [57], [58], [59]. In addition, combined exposure to multiple pesticides can have additive or synergistic adverse effects in bees [60], [61], [62], [63] with eventually underestimated consequences [64]. Similarly, multifactorial impact arising through combined chronic exposure to pathogens and pesticides may trigger detrimental feedbacks of supposedly sublethal individual stressors at the colony level that could explain otherwise enigmatic negative impacts [65]. In this regard, an important but yet poorly understood aspect represents the adverse influence of neonicotinoids on the honeybee’s immune system [66], [67]. There is growing evidence for detrimental interactions and compromised immunity in honeybees upon combined exposure to neonicotinoids and pathogens, including the prevalent gut-parasite *Nosema* spp. [36], [68], [69], [70], [71] and near-ubiquitous viruses, such as the typically *V. destructor*-associated deformed wing virus [72] or the black queen cell virus [36]. To date it remains unclear to what extent these findings can be extrapolated to field settings where, for example, immunity-related patterns in honeybees may be strongly influenced by many more environmental factors, such as the overall nutritional status [24], [73], [74], [75], [76]. While several unplanned field exposures resulted in massive effects of neonicotinoids under certain circumstances [41], [77], [78], there is no compelling evidence so far that field-realistic neonicotinoid exposure resulting from standard agronomic implementations of neonicotinoids pose a serious threat to whole colonies [79], [80], [81], [82], [83]. Nevertheless, some regulatory bodies reacted recently [84], [85] in order to clarify whether the overall contrasting results may have been influenced by: overestimating individual predictors while dynamics at multiple levels were neglected [65]; by the lack of statistical power for individual studies [44]; or by the possibility that especially sensitive endpoints have simply been missed. For instance, the long-term impacts of neonicotinoids, which might only become evident during sensitive phases like overwintering, have received little attention to date. Moreover, information on how neonicotinoids could impact on queens is virtually lacking [64]. It is unknown whether queens are relatively protected from agricultural pesticides through receiving processed food from hive bees only, or whether trophallactic interactions with the usually most long-lived honeybee in a hive could indeed represent a sink for trace residues of such systemic compounds. Sublethal pesticide exposure could have important consequences for colony fate through compromising the queen’s cognitive abilities or immune system and thereby reducing her performance. For instance, it is known that replacement of failing queens by the worker bees, i.e. queen supersedure, can be triggered by reduced oviposition of old or insufficiently mated queens [86], [87]. Colony fitness is another sensitive but largely neglected aspect of honeybee colony performance. In bumblebees, for example, it has repeatedly been shown that the negative impact of chronic neonicotinoid exposure on individuals and colony performance was less pronounced compared to queen production [88], [89], [90]. Similarly alarming fitness effects upon chronic sublethal neonicotinoid exposure are indicated in solitary bees [91]. Reproductive success, however, is vital for inferring long-term population level consequences. Compared to the assessment of much more general traits of colony performance and productivity in honeybees, the quantification of fitness in the true sense is very difficult because of the complex socio-biology of reproduction in honeybees. In managed honeybee colonies, besides male mating success, swarming can be considered as a tangible proxy of fitness [92], which is in practice primarily linked to beekeeping management decisions though.

Here we experimentally assessed the effects of chronic dietary neonicotinoid exposure on honeybee colony performance and fitness on a temporal scale and in relation to the honeybees’ genetic background. In a fully crossed experimental design 24 freely flying honeybee colonies, including two groups of sister-queens from different strains (14 and 10 colonies, respectively), were placed at a single apiary and were either exposed to control pollen or pollen that has been spiked with a combination of the two neonicotinoids thiamethoxam and clothianidin (on average 5.31 µg/kg and 2.05 µg/kg, respectively) via in-hive feeding over two brood cycles (see Figure S1). All colonies were then identically maintained and controlled against *V. destructor* throughout a one year monitoring period. During the study four detailed colony assessments, including estimates of numbers of adult bees and the amounts of brood and stores, were conducted to evaluate potential effects on colony performance and productivity in the short- (1.5 months), medium- (3.5 months) and long-term (1 year). We used this experimental design in order to contribute to better understand three currently poorly resolved aspects. First, while the majority of experiments at the colony level applied sublethal chronic neonicotinoid exposure through sucrose solution, we hypothesized that neonicotinoid-contaminated pollen provides stronger exposure of larval stages and nurse bees that may express sublethal effects when performing more complex tasks in later life cycle stages [53], [93], thereby potentially resulting in delayed effects on colony performance. Second, contaminated pollen could result in sublethal exposure of honeybee queens that may affect their performance and subsequently cause failure. Therefore, we assessed the fate of queens one year subsequent to experimental feeding of neonicotinoid-spiked pollen, as well as the colonies’ propensity to swarm as an indicator for colony fitness. Third, we addressed the question of whether neonicotinoid susceptibility at the honeybee colony level has a genetic basis.

## Materials and Methods

### Experimental setup and colony maintenance

Twenty-four honeybee colonies were established using artificial swarms (1.5 kg of bees) in summer 2010. Two groups of sister-queens originating from different, locally adapted breeding populations were introduced in order to control for the honeybees’ genetic background and maternal effects: one group of 14 queens from a region in eastern Germany that is characterized by intense agriculture, and one group of 10 queens from an alpine region in central Switzerland. All queens were freely mated at apiaries in corresponding geographic regions during early summer 2010 and then individually tagged and clipped one forewing. The former group of colonies represented *A. m. carnica*, whereas the latter represented predominantly *A. m. mellifera*, and in the following they are referred to as strain A and B, respectively.

As permitted by the veterinary agency of the canton Zurich, we established an apiary on the land of the research station Agroscope Reckenholz-Tänniken in a rural area near the city of Zurich, Switzerland (47°25′38N 8°31′11E). Two groups of each 12 hives were placed in a single row, with all hive-entrances pointing in the same direction. To meet a fully-crossed experimental design, artificial swarms containing queens of the different strains were randomly allocated to the two experimental groups (N = 7 from strain A and N = 5 from strain B each). Hives containing queens of the two different strains A and B were ordered identically in each experimental group (see Figure S1 for details of the setup). Hives within groups were separated by 1 m, and both groups of hives were separated by approximately 20 m distance, including shrubs as landmarks to minimise forager drift between groups [94]. All colonies were equipped with brand new hive material, including polystyrene hive bodies, wooden frames (200×350 mm in size) and organically certified, pesticide-free wax foundations. Commercial pollen traps (Wienold, Lauterbach, Germany), painted in different colours, were installed at all hive entrances (identical colour sequence in each group), but only activated during the exposure phase (see below). All colonies were identically treated against *V. destructor* using oxalic acid (40 ml sucrose solution containing 3.5% w/w oxalic acid per colony) five days after establishment of artificial swarms (in the absence of capped brood), and fed with commercial sugar syrup (sugar beet based, 73% sugar content, containing equal proportions of glucose, fructose and sucrose; Hostettler’s, Zurich, Switzerland) during summer 2010 to promote colony growth. Pollen was assumed to be available in sufficient quantities. All colonies overwintered on eleven combs, and oxalic acid treatment against *V. destructor* was repeated in December 2010.

During spring and summer 2011 colonies were not fed but left to freely collect nectar and pollen. All colonies were simultaneously provided with a second and third hive body containing 11 frames with wax-foundations for comb building in early April 2011 and in mid-May 2011, respectively. The upper hive body provided last was separated by a queen excluder to ensure honey storage only, and on the same day it was provided the experimental treatment was initiated and lasted until end of June 2011 (see below). In mid-July 2011 colonies were taken off their honey combs and subsequently maintained on 22 combs. They received 12.5 kg of sugar syrup during late July and late August 2011 (25 kg in total). After each feeding phase, colonies were simultaneously treated against *V. destructor* using 130 ml of formic acid (70% w/w) evaporating from commercial dispensers (Andermatt Biocontrol AG, Grossdietwil, Switzerland) for about one week each during early August and early September 2011. Colonies were then overwintered and treated with oxalic acid in December 2011 (see above). At no point during the study synthetic acaricides were used for varroa mite management. Subsequent to overwintering colonies were monitored until June 2012 without further intervention. No honey supers were provided in 2012 in order to increase the propensity for swarming, which served as a proxy of fitness one year after the treatment (see below).

### Treatment procedures and residue analyses

In mid-May 2011 pollen traps were activated to vastly prevent pollen inflow, and in-hive pollen feeding was initiated. Pollen patties consisted of 55% honeybee pollen (common stock of commercial pollen with mixed floral content of at least 19 plants; Sonnentracht Imkerei, Bremen, Germany), 5% brewer’s yeast and approximately 40% sucrose (two thirds 73% sugar syrup and one third powder sugar). Three times per week (each Monday, Wednesday and Friday) all colonies were provided with two 200 g pollen patties loosely packed in cellophane paper and placed between the two lower hive bodies (i.e. within the brood nest). Disturbance of the colonies was thus reduced to a minimum. The bees easily corroded the cellophane paper to access the content. Two pollen patties of 200 g were generally consumed completely within 48 hours by each colony. Prior to feeding, pollen was gamma ray irradiated (Leoni Studer Hard AG, Däniken, Switzerland) to prevent putative pathogen spill over (e.g., see [95]). One group of 12 colonies (see above) received plain pollen while the other received patties containing environmentally relevant residues of the two neonicotinoids thiamethoxam and clothianidin (see below). Chronic neonicotinoid exposure through in-hive pollen feeding was performed for 46 days (1.5 months) in order to cover two brood cycles, thereby resulting in total provisions of 8 kg of pollen patties per colony. Pollen traps were then deactivated to no longer prevent colonies from storing pollen collected outside.

For both neonicotinoids pure analytical standards (PESTANAL, Fluka; with purities of 99.7 and 99.9% for thiamethoxam and clothianidin, respectively) were purchased (Sigma-Aldrich, Seelze, Germany), dissolved in distilled water (1 mg/L) and then stored at room temperature and protected from light. Aliquots of a single stock solution for each parent compound were added to the sucrose solution, which was then thoroughly mixed into the pollen and yeast. A commercial kneader was used to produce a homogenous paste to be portioned in cellophane paper (200 g) and kept frozen until usage. In total, 20 mixtures of plain and neonicotinoid-spiked pollen were prepared and fed batch-wise over time. A subsample of each of these batches of pollen patty preparations was subjected to residue analyses performed by the United States Department of Agriculture, Agricultural Marketing Service, Science and Technology Laboratory Approval and Testing Division of the National Science Laboratories in Gastonia, North Carolina. All samples were extracted for analysis of agrochemicals using a refined methodology for the determination of pesticides using an approach of the official pesticide extraction method (AOAC OMA 2007.01) using an acetonitrile:water solution and analysed by liquid chromatography coupled with tandem mass spectrometry detection (LC/MS-MS) utilising the parent and confirmatory ions of thiamethoxam and clothianidin. Samples were analysed using certified standard reference materials for the presence of both compounds with a limit of detection of 4.0 ppb for thiamethoxam and 1.0 ppb for clothianidin. Our target concentrations were 5.0 and 2.0 ppb for thiamethoxam and clothianidin, respectively. Since clothianidin is the major metabolite of thiamethoxam [96], [97], both bioactive compounds will co-occur in the pollen and nectar of thiamethoxam-treated crops and were therefore applied in combination. The concentrations used here match field-realistic levels of both compounds previously found in pollen of treated crops [41], [42], [46], [80], [82], [98]. In order to confirm the absence of unexpected additional exposure, we also subjected six random samples of the sugar syrup used for late season feeding (2010 and 2011), six samples of the original pollen stock, as well as four pollen patty samples of the control group to residue analyses. Moreover, pollen trap contents collected during the experimental feeding were pooled across colonies of each experimental group and samples from five collection dates throughout the treatment were taken for residue analyses. Additional matrix endpoints at the end of the experimental pollen feeding phase for both treatment groups were forager bees (1 sample pooled across all colonies) and pupae close to adult emergence (3 samples pooled across 4 colonies each). Furthermore, in both treatments we sampled wax (2 samples pooled across 6 colonies each) and bee bread (4 samples pooled across 3 colonies each) 3 weeks after the pollen treatment when honey supers were removed, as well as one sample of honey from each colony of the neonicotinoid-exposed group (pooled from at least 3 different combs). All samples for residue analyses were stored frozen and shipped on dry ice.

### Population estimates and data collection

Estimates of colony strength were performed using the ‘Liebefelder Method’ [99], [100]. Specifically, we visually estimated the number of adult bees, the amount of capped brood (pupae) and un-capped brood (eggs and larvae), and the amount of honey and pollen stores in dm^2^. Colony assessments were consistently performed by the same person and alternated between treatment groups during the day. During each colony assessment the presence of the original queen was checked. Successful queen replacement was counted as the presence of a non-tagged, egg-laying queen which possessed two intact forewings, either in the presence or absence of the originally tagged mother-queen. Based on 1 dm^2^ comb containing 400 cells on average, the estimated proportion of comb area comprising of brood was converted into numbers of individuals in order to treat corresponding response variables as counts, whereas estimates of honey stores were converted into total weight (based on an average weight of 2 kg for fully filled honey combs of the used frame format).

In spring 2011 (mid-May) the first colony assessment was performed and three days later the experimental treatment was initiated. During the pollen feeding phase, contents of all pollen traps were collected and weighed each time when new pollen patties were provided, resulting in 20 pollen collection records per colony in total. In summer 2011 (beginning of July), two days after the last pollen patties were fed, the second colony assessment was conducted to evaluate short-term effects on colony performance. After the exposure phase the control and the neonicotinoid-treated colonies were maintained identically and the third colony assessment was performed 3.5 months after the exposure in autumn 2011 (mid-October), to evaluate medium-term effects colony performance before overwintering. Overwintering success was assessed end of March 2012 and thereafter surviving colonies were inspected on a weekly basis. Finally, the fourth colony assessment for all colonies that survived winter was performed one year after the treatment in spring 2012 (late April) to evaluate long-term effects on colony growth. Afterwards colonies were maintained until June 2012 and inspected for queen cells on a weekly basis and for swarming events at least every second day. Since original queens had one clipped forewing, swarms remained nearby the hive and could thus be easily recognized.

### Statistical analyses

To investigate the effects of exposure to thiamethoxam and clothianidin on honeybee colony performance over time, we analyzed a set of sensitive endpoints within the framework of mixed models.

#### Model formulation and selection

The response variables (endpoints) were numbers of adult honeybees, pupae and eggs and larvae. These were modelled including the explanatory variables (factors) treatment (control and neonicotinoids), honeybee strain (A and B), and assessment date (spring 2011, summer 2011, autumn 2011 and spring 2012) as fixed effects, and colony as a random effect. Residual analysis of all response variables indicated the need for variance stabilization and variables were transformed accordingly. The data for the endpoints number of adult honeybees and pupae were square-root transformed. Residuals for eggs and larvae displayed a more complex pattern of a bow-shaped variance being largest for medium fitted values (∼8000) and decreasing for larger and smaller fitted values. This pattern resembles that of a binomially distributed variable, which conforms well to the upper bounded egg-laying rate of a honeybee queen. In such cases effective counts of a given response variable can be divided by the expected maximum number to obtain binomially distributed variables. Based on Khoury *et al*. [101], we set the limit to a daily egg-laying rate of 2000 and, according to the honeybee life-cycle, 16000 eggs and larvae present at any time. Ratios of actually present and maximum possible numbers of eggs and larvae were arcsine square-root transformed to stabilize variances for further analyses, as commonly performed for binomially distributed variables.

Numbers of adult honeybees exhibited an increased variance at the fourth assessment date in spring 2012, which prompted us to use a weighted linear mixed model for this response variable. Weights were set as the inverse of the residual variances of the two groups (for the assessments between spring and autumn 2011 and the assessment in spring 2012, respectively).

For each endpoint, complete models were fitted based on the threefold interaction term of the explanatory variables (fixed effects) plus the random effect. Model simplification was evaluated by hierarchically removing interaction terms based on likelihood ratio tests. The goodness of corresponding Chi-squared approximations was confirmed by model based parametric bootstrapping. During all steps of the model selection, model assumptions and serial correlations of the residuals of colonies were inspected.

#### Hypotheses testing with contrasts

To test for effects of neonicotinoid exposure on colony performance and the influence of the honeybees’ genetic background on responses to the neonicotinoid-treatment, one-sided contrasts and corresponding *P*-values (adjusted for multiple testing) were computed for the overall treatment effect at each individual assessment date and, when the threefold interaction significantly contributed to model a given response, also for treatment nested within honeybee strain. Since seasonal effects were expected, contrasts including assessment date were not performed.

#### Further statistical analyses

To investigate effects of chronic exposure to thiamethoxam and clothianidin on honey production, the difference of the log transformed total weights before and after the experimental pollen feeding was analysed using linear regression. The full model was fitted with neonicotinoid exposure and honeybee strain as fixed factors and the interaction between them. In the same way we compared comb areas in dm^2^ comprising of pollen stores (bee bread) in the hives before and after the experimental pollen feeding in order to evaluate pollen consumptions during the treatment.

The time series of twenty pollen collections per colony sampled during the 1.5 months of experimental in-hive pollen feeding were converted into respective Areas Under the Curve (AUC) for further analysis. The AUC represents the overall colony-specific pollen foraging activity, with higher AUC values corresponding to higher collection performances. We analyzed AUC with a one-sided Mann-Whitney test for a difference between neonicotinoid-exposed and control colonies.

Supersedure of original queens (at any time) and swarming events in spring 2012 yield a yes/no value for each colony. The associations of these variables with neonicotinoid treatment were investigated using Fisher’s exact tests for contingency tables of small sample sizes.

All statistical analysis were performed using R [102]. Mixed models were fitted using the lmer function of the *lme4* package [103], and contrasts were performed using the glht function of the *multcomp* package [104].

## Results

### Residue analyses

Original stocks of honeybee pollen and sugar syrup used to prepare pollen patties in our experiment did not contain traceable amounts of the two neonicotinoids thiamethoxam and clothianidin, even when the limit of detection was reduced to 0.1 ppb for both compounds. Thus, thiamethoxam and clothianidin are considered being absent in the pollen patties fed to the control colonies. In contrast, in all samples from the pollen patties that have been spiked with the two neonicotinoids both parent compounds were detected in the range of the target concentrations: The effective mean concentrations (± SD across different pollen patty batches) used during the neonicotinoid treatment were determined to be 5.31±0.75 ppb for thiamethoxam and 2.05±1.18 ppb for clothianidin. The residue analyses document constant chronic exposure to thiamethoxam and clothianidin at field-realistic levels in pollen over a period of two honeybee brood cycles. There was no unexpected additional exposure to thiamethoxam or clothianidin from the outside, as indicated by the lack of traceable residues of both compounds in the pollen collected from pollen traps of both treatment groups during the in-hive pollen feeding phase. In none of the forager bee and pupae samples collected directly after the treatment and in none of the hive samples collected 3 weeks after the treatment (i.e. bee bread, honey and wax) residues of either compound above respective limits of detection could be found in both the control and neonicotinoid treatment. The absence of residues in bees is not surprising given the low concentrations used here, yet the absence of in-hive residues 3 weeks after feeding contaminated pollen over two brood cycles indicates that low level residues may quickly disappear in the hive matrix (see also [80], [82]) through consumption, dilution or degradation.

### Colony growth

In the control group and in the group exposed to thiamethoxam and clothianidin each 1 colony of strain A lost their queen after the pollen feeding phase during the formic acid treatment against varroa mites in 2011. Moreover, in each experimental group 1 colony became queenless during winter (originating from strain B and A in the control and in the neonicotinoid-exposed group, respectively). Thus, mixed model analyses are based on 12 colonies per experimental group for the first and the second colony assessment (in both treatment groups 7 colonies originating from strain A and 5 from strain B), while 11 colonies per group were available for the third colony assessment (in both treatment groups 6 colonies originating from strain A and 5 from strain B), and 10 colonies were available for the fourth colony assessment (in the control 6 and 4 from strain A and B, respectively, and for the neonicotinoid treatment each 5 from strain A and B). Queenless colonies were removed immediately after queen loss was recognised.

**Figure 1:**
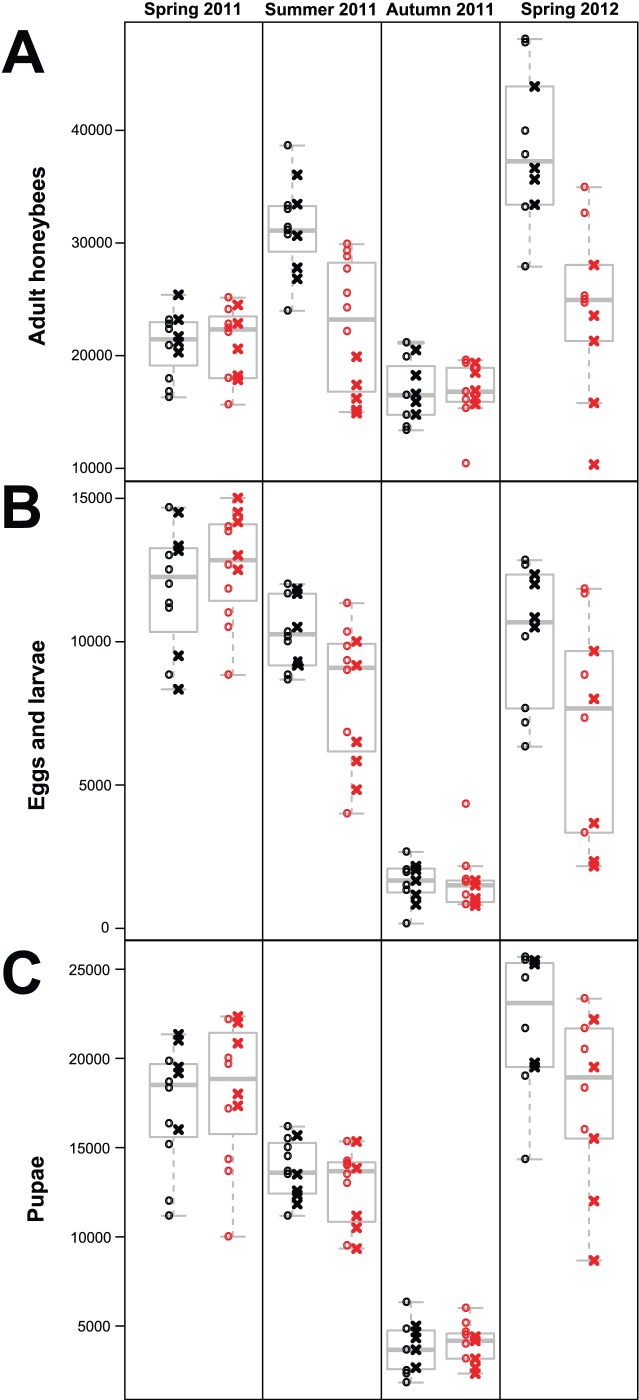
Dynamics of honeybee colony performance. Data of all three endpoints number of adult bees (A), eggs and larvae (B) and pupae (C) for the different pollen feeding treatments (black  =  control; red  =  neonicotinoids) and honeybee strains (circles  =  strain A; crosses  =  strain B). The data were obtained at four successive colony assessment dates (X-axis subpanels within figures) performed before (Spring 2011) and directly after the 1.5 months of experimental pollen feeding (Summer 2011), 3.5 months after the treatment (Autumn 2011) and one year later (Spring 2012). Estimated numbers on the Y-axes are truncated for adult bees and pupae for better overview.

The data for the three endpoints adult bees, pupae and eggs and larvae across assessment dates are summarized in Fig. 1A–C. Dynamics of colony strength and brood curves displayed the expected general pattern of seasonal variation. However, we detected strong effects of neonicotinoid exposure, as well as interactions of the honeybees’ genetic background with neonicotinoid exposure. The number of adult bees and the number of eggs and larvae were each best explained by the threefold interaction term model, i.e. neonicotinoid exposure, honeybee strain and assessment date, while for the number of pupae the retained model included the twofold interactions of assessment date with honeybee strain and neonicotinoid exposure, respectively, but no threefold interaction. Model-based estimates of contrasts and corresponding significance levels are summarized in Table 1. After 1.5 months of experimental pollen feeding, there was a significantly negative influence of the exposure to thiamethoxam and clothianidin on the number of adult bees for both honeybee strains, whereas this effect was much stronger for honeybee strain B than for strain A (Fig. 1A). Overall, average worker populations were 28% smaller in the neonicotinoid treatment compared to the control. There was also a significant overall decrease of the number of eggs and larvae in the neonicotinoid treatment, yet, this effect was not significant when tested within either honeybee strain A or B (Fig. 1B). However, there was no significant effect of nenicotinoid treatment or honeybee strain on the amount of pupae after the experimental pollen feeding (Fig. 1C). Compared to the control, the average amount of total brood had declined by 13% in the colonies exposed to thiamethoxam and clothianidin. No effects of the previous neonicotinoid exposure on the amount of adult honeybees or honeybee brood were detected 3.5 months after the experimental pollen feeding (Fig. 1A–C). Interestingly though, one year after the experimental pollen feeding, the negative impact of the previous neonicotinoid treatment on the number of adult bees was even stronger than directly after exposure to thiamethoxam and clothianidin (Fig. 1A). These effects were significant within honeybee strains A and B, but again much more pronounced in strain B. Similarly, when the overall significant decrease of the amount of eggs and larvae one year after the neonicotinoid treatment was tested within strains, a significant effect was only detected for honeybee strain B (Fig. 1B). Moreover, contrasting to the finding directly after exposure to thiamethoxam and clothianidin, there was also a significantly detrimental effect of the neonicotinoid treatment in the previous year on the amount of pupae (independent of honeybee strain), see Fig. 1C and Table 1.

**Table 1:** Model-based estimates of contrasts and corresponding significance levels of the treatment effect (neonicotinoid *versus* control) and honeybee genetics (strain A *vs.* strain B).

	Adult bees			Eggs and larvae			Pupae		
	Summer2011	Autumn2011	Spring2012	Summer2011	Autumn2011	Spring2012	Summer2011	Autumn2011	Spring2012
Neonicotinoids *vs* Control	−60.56***	−0.73	−82.96***	−0.31*	−0.01	−0.49***	−4.36	1.84	−15.31**
Strain A *vs* strain B							5.31	2.65	7.91
Treatment within strain A	−14.07*	−1.33	−28.59*	−0.10	0.03	−0.06			
Treatment within strain B	−46.49***	0.59	−54.37***	−0.21**·**	−0.05	−0.42***			

Results are shown in the transformed scale for the three response variables adult bees, eggs and larvae and pupae assessed directly after the 1.5 months of treatment (Summer 2011), 3.5 months later (Autumn 2011) and 1 year later (Spring 2012). For adult bees and eggs and larvae (the models that included a significant threefold interaction between treatment, honeybee strain and assessment date) contrasts for treatment effects were also computed within individual honeybee strains at each assessment date. *P* values are adjusted for multiple testing. ***P<0.001; **P<0.01; *P<0.05; **·** 0.05<*P*<0.1.

### Honey production

At treatment initiation all colonies already harboured considerable honey stores due to comparatively early spring flowering in 2011. Strain A and B, respectively, had on average 24.8±2.9 and 27.0±2.9 kg in the control group, and 25.4±2.8 and 23.6±0.7 kg in the group subsequently exposed to thiamethoxam and clothianidin. During the experimental pollen feeding, honey stores in the control group increased by 7.7±4.4 kg per colony on average (10.3±3.6 and 4.1±2.2 kg for colonies of strain A and B, respectively). During the same period honey stores in the neonicotinoid-exposed group also slightly increased for colonies of strain A (1.8±2.1 kg on average), but decreased for neonicotinoid-exposed colonies of strain B (−4.9±1.0 kg on average), resulting in an overall decrease of −1.0±3.8 kg per colony on average. Honey production during the treatment was significantly influenced by both neonicotinoid exposure and honeybee strain: While strain B was significantly less productive than strain A independent of the treatment (*F*_1,21_ = 40.40, *P*<0.001), neonicotinoid exposure negatively affected honey production in both strains (*F*_1,21_ = 68.18, *P*<0.001). The interaction between both predictors was not significant (*F*_1,20_ = 1.45, *P* = 0.24) and was thus removed prior to testing the main effects. Overall, the mean honey production over the entire season (including honey production during the pre-treatment phase) remained 29% lower in the neonicotinoid-exposed colonies (23.7±2.5 kg) compared to the control (33.4±5.1 kg).

### Pollen consumption

At treatment initiation, pollen stores (bee bread) in the control group comprised of 17.9±10.7 dm^2^ and those in the group subsequently exposed to thiamethoam and clothianidin comprised of 20.8±3.8 dm^2^. After the experimental pollen feeding pollen stores within hives comprised of 29.8±8.9 dm^2^ and 24.9±11.7 dm^2^ in the control and neonicotinoid-exposed group, respectively. There was no indication that pollen storing and pollen consumption, respectively, during the experimental pollen feeding was influenced by neonicotinoid exposure (*F*_1,21_ = 2.63, *P* = 0.12) or honeybee strain (*F*_1,21_ = 2.52, *P* = 0.13). The interaction between the two predictors was not significant (*F*_1,21_ = 0.74, *P* = 0.40) and was thus removed prior to testing the main effects.

### Pollen collections

**Figure 2:**
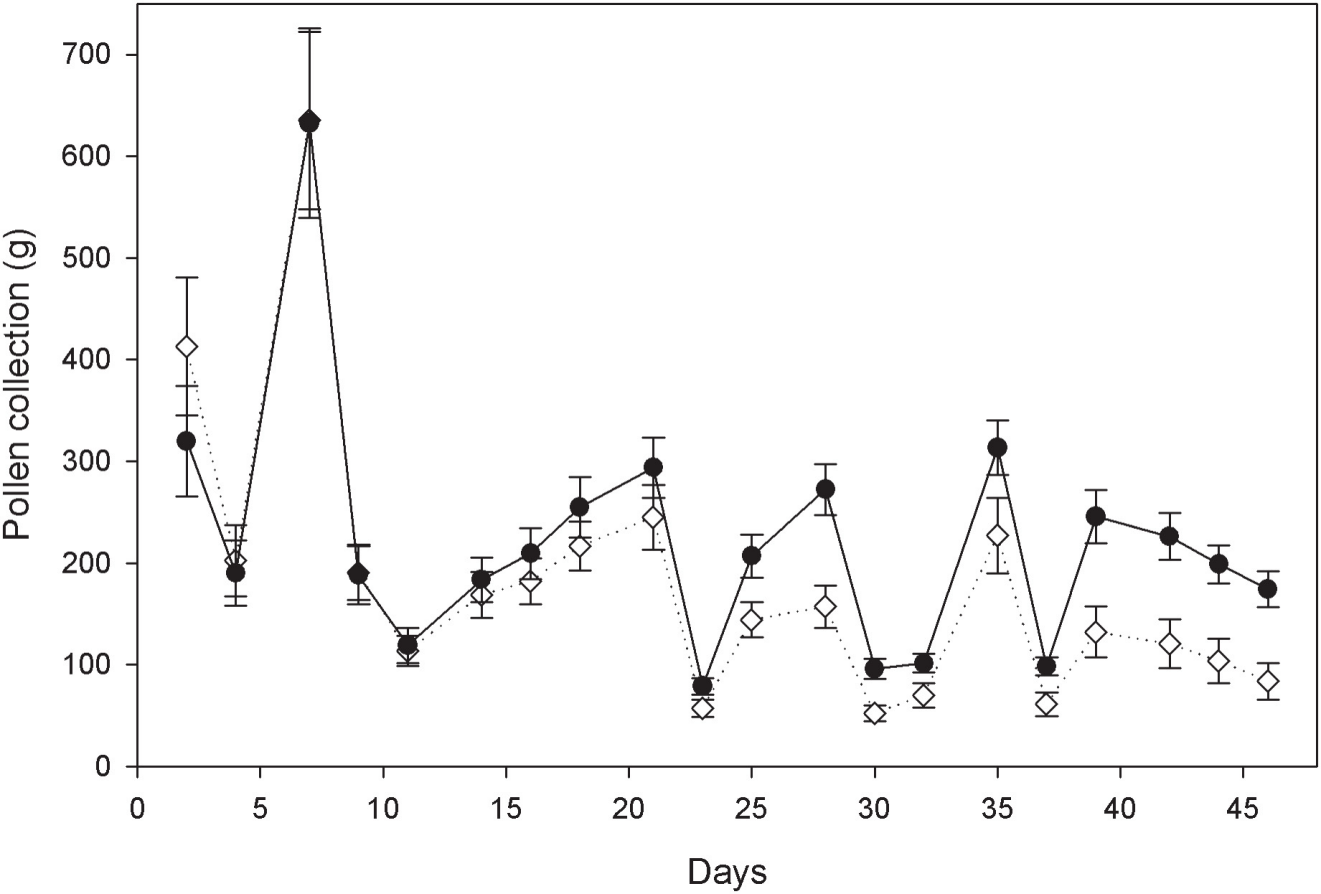
Pollen collections. Mean (±SD) fresh weights of pollen collections for control (black) and neonicotinoid-exposed (white) colonies over the course of the treatment period (pollen-trap contents were weighed in 2-2–3 days intervals throughout the study).

Total mean pollen harvests (±SD) per colony, as inferred from pollen trap contents, were 4.4±0.47 kg and 3.58±0.43 kg for the control and neonicotinoid-exposed group, respectively. The colonies exposed to thiamethoxam and clothianidin had 19% lower total pollen collections on average. Pollen collections measured as AUCs were found to be significantly lower in neonicotinoid-exposed colonies (*P*<0.001). While both treatment groups collected similar amounts of pollen during the first 3 weeks of the experimental pollen feeding, colonies exposed to thiamethoxam and clothianidin consistently collected less pollen later on, with mean pollen collections barely reaching more than 50% of the control group during the last week of exposure (Fig. 2).

### Supersedure of queens and tendency to swarm

We found a significant association of neonicotinoid exposure and queen supersedure (in the absence of swarming) (*P* = 0.01): while all ten queens of the control group survived until the end of the experiment (∼2 years or swarmed, see below), 6 out of 10 queens of the colonies experimentally exposed to thiamethoxam and clothianidin over 1.5 months were replaced within one year after treatment. The result remained significant when overall queen loss was assessed, i.e. also including the two colonies (one per treatment group) that lost their queen during winter (*P* = 0.02). A negative association of neonicotinoid exposure and swarming events during spring following experimental treatment was found (*P* = 0.005): in the control group 9 out of 10 colonies swarmed until end of May 2012 (5 out of 6 colonies of strain A and all 4 colonies of strain B), while only 2 colonies (one of strain A and B each) of the group that was exposed to thiamethoxam and clothianidin in the previous season swarmed.

## Discussion

The major findings of this study using sublethal chronic exposure of honeybee colonies to thiamethoxam and clothianidin through feeding contaminated pollen were: (*i*) significant short-term (1.5 months) impacts at the colony level resulting in decreased colony performance and productivity; (*ii*) no negative influence in the medium-term (3.5 months) and on colony overwintering; (*iii*) significantly decelerated colony growth in the long-term (1 year) that was associated with higher queen supersedure rates and a reduced tendency to swarm; and (*iv*) significant interactions of the honeybee genetics with the observed effects of neonicotinoids on most parameters. In the following, these findings are discussed in context.

### Short-term impact

At the colony level, honeybee foraging efficiency was negatively influenced during chronic exposure to pollen containing environmentally relevant concentrations of the two neonicotinoids thiamethoxam and clothianidin over two brood cycles (1.5 months). The detected decrease in pollen collection and honey production upon sublethal neonicotinoid exposure are in line with earlier findings of impaired honeybee foraging through impacts on neurophysiological traits and cognitive abilities, including sucrose responsiveness, foraging rates, waggle dancing and memory and learning [47], [48], [49], [50], [51], [52], [53], [54], [55], [56], [57], [58], [59]. In addition, increased forager losses resulting from decreased homing success [48] through compromised navigation memory [58] may have contributed to both reduced foraging efficiency and significantly smaller worker populations of the colonies exposed to thiamethoxam and clothianidin compared to controls (Fig. 1A, Table 1). Indeed, there were no significant effects on the numbers of pupae present at the end of the exposure phase (Fig. 1C, Table 1), which points at higher forager losses rather than decreased worker production of neonicotinoid-exposed colonies during the treatment. As opposed to known effects of neonicotinoid ingestion of foragers through nectar-substitutes [47], [48], [49], [50], [51], [56], [57], [58], the overall reduced numbers of adult bees through pollen exposure is intriguing. Pollen is generally not stored extensively within hives but consumed quickly. There was no evidence for differences in pollen storing between treatment groups directly after the experimental feeding. Therefore, it can be assumed that control pollen and pollen spiked with thiamethoxam and clothianidin were similarly consumed within experimental colonies. Contaminated pollen may affect various life stages of honeybees [105], yet pollen is predominantly consumed by nurse bees and larvae, but not foragers. In this regard, the recent finding that sublethal neonicotinoid exposure of honeybee larvae impacts post-emergence olfactory associative behaviour of adults [53] is relevant, and might be one of the largely unresolved effects arising from altered larval gene expression profiles upon sublethal neonicotinoid exposure [93] that may help to explain the strong impediment of colony growth documented here. Delayed sublethal effects extending to later life-cycle stages could similarly apply to larvae and nurse bees consuming contaminated pollen [64] and, depending on colony exposure duration, be able to reinforce decreased foraging efficiency. Our observed pattern of increasing divergence of pollen collections between treatment groups after 2–3 weeks, which roughly corresponds to the adult in-hive phase after emergence [106], may be indicative of the recruitment of less efficient foragers, which have encountered pollen contaminated with thiamethoxam and clothianidin during their nursing phase. The successively greater decline of pollen collections of neonicotinoid-exposed colonies after 5 weeks, in turn coincides with the time frame during which foragers could be expected to be exposed to contaminated pollen from the young larva stage onwards and throughout their entire development. Further research is needed to evaluate the potential for delayed impact on implementing complex foraging tasks through altered metabolic networks caused by sublethal dietary neonicotinoid exposure to honeybee brood [53], [93] and eventually also nurse bees [64], the developmental stages during which fundamental physiological processes required for adult olfaction and learning performance are settled [107].

The observation of strongly reduced pollen collection of the colonies exposed to thiamethoxam and clothianidin during the last week of the treatment could also be interpreted as a response to significantly reduced numbers of larvae (Fig. 1B, Table 1). However, a general decrease in foraging efficiency is also indicated by significantly reduced honey production. Because experimental pollen was provided constantly in sufficient amounts, significantly reduced investment in rearing larvae after 1.5 months of combined exposure to thiamethoxam and clothianidin is best explained by the declining numbers of workers: higher forager losses trigger premature forager recruitment, which results in fewer nurse bees available for brood rearing [101], [108]. Although physiological changes in nurse bees may have played an additional role [54], [55], [93], [109], reduced investment in brood rearing was probably reinforced by the reallocation of worker resources at the colony level towards the end of the exposure period rather than by adverse effects on the larvae themselves. Although there is some indication for negative effects of neonicotinoids and other pesticides on honeybee larvae [62], [93], [110], there is so far no evidence that field-realistic neonicotinoid exposure in the absence of additional stressors results in increased larval mortality [36], [64]. The latter would also be difficult to reconcile with the observation that the numbers of pupae did not differ between treatments, even after 1.5 months of neonicotinoid exposure. Interestingly, short-term effects of the combined exposure to thiamethoxam and clothianidin on the number of adult bees were influenced by the honeybees’ genetic background. While effects on colonies of strain A were only marginally significant, those on colonies of strain B were highly significant (Fig. 1, Table 1).

### Medium-term impact

The colony assessment during autumn 2011, 3.5 months after the experimental pollen feeding, revealed that there were neither effects of the combined exposure to thiamethoxam and clothianidin nor of the honeybees’ genetic background on colony strength and the amount of brood (Fig. 1A–C, Table 1). All colonies were strong and well-fed, and thus overwintered successfully, except one colony of each treatment which lost their queens during winter. This reiterates the general view that a sustainable varroa mite management is a major aspect of honeybee colony health, thereby limiting colony losses [25], [26]. Our comprehensive varroa mite management, comprising of a combination of integrated actions using organic acids [27] but avoiding potentially detrimental synthetic acaricides [47], [52], [111], [112], was apparently sufficient to limit *V. destructor-*associated damage during the sensitive overwintering period, and throughout the entire study. A thorough varroa mite control pre-supposed, the overcoming of the previously observed short-term effects of neonicotinoids on colony strength and brood rearing 4–5 brood cycles later shows that chronic sublethal neonicotinoid-exposure alone does not trigger elevated honeybee colony winter losses [17], [20], although contrasting results have been found in other studies [113], [114].

There were no obvious clinical symptoms of infections with widespread honeybee pathogens such as the ectoparasitic mite *V. destructor*, microsporidian gut parasites *Nosema* ssp., chalkbrood (the fungal pathogen *Ascophaera apis*) or European and American foulbrood (bacterial diseases caused by *Melissococcus plutonius* and *Paenibacillus larvae*, respectively) at any time during the study. Nevertheless, it cannot be excluded that some of the above described short-term effects on colony performance may have been influenced by detrimental neonicotinoid-pathogen interactions at the level of individual bees [36], [68], [69], [70], [71], [72]. Higher forager losses associated with overall shifts in forager age structure may also cause generally decreased immunological competence at the colony level [115]. However, these differentiations between individual drivers and their interactions are only of limited practical importance because free-flying honeybees will almost inevitably carry ubiquitous pathogens and thus encounter combined pressures. In fact, it is very likely that latent infections with the most common *V. destructor*-associated honeybee viruses and *Nosema* spp. have also been present in the experimental apiary. The common garden approach to maintain all colonies at the same isolated field site for almost one year prior to the experiments actually aimed at ‘synchronizing’ viral and microbial landscapes across colonies, thereby resulting in potential errors of systematic conservative nature. If multiple pressures were indeed present in our experiment, the observed recovery from decreased performance of previously neonicotinoid-exposed colonies after several brood cycles conforms to model predictions, showing that even detrimental interactions presumably can be tolerated to some extent and do not necessarily result in disrupting colony function [65].

### Long-term impact

Given that significantly negative short-term impacts of combined exposure to thiamethoxam and clothianidin during summer 2011 had faded with respect to colony strength during autumn (medium-term) and overwintering success, the patterns observed during the subsequent spring were unexpected. Colonies that received pollen spiked with thiamethoxam and clothianidin during the previous spring exhibited significantly lower numbers of adult bees compared to controls one year later, whereas the effects were much stronger for strain B than for strain A (Fig. 1A, Table 1). In addition, there were significantly negative overall effects of last year’s neonicotinoid exposure on the amount of pupae (Fig. 1C, Table 1) and eggs and larvae (Fig. 1B, Table 1). For the latter endpoint, however, significant effects were only found for strain B (Table 1). These patterns of decelerated colony growth are intriguing, because all hive samples collected 3 weeks after the experimental feeding of neonicotinoid-spiked pollen patties did not contain traceable residues of thiamethoxam and clothianidin. Although trace residues below corresponding limits of detection could have still been present within hives one year after the treatment, it is highly unlikely that this explains detrimental effects that far exceeded those observed directly after the treatment (Table 1). Instead, reduced performance of previously neonicotinoid-exposed queens appears to be a more plausible explanation for long-term effects. While all original, individually tagged and wing-clipped queens were recognized in autumn 2011, the assessment in spring 2012 revealed that there were non-tagged egg-laying queens with intact wings in some colonies previously exposed to thiamethoxam and clothianidin. This clearly shows queen supersedure during the previous autumn as a response to poor queen performance [86], [87], which could pose a risk for successful overwintering. Yet, supersedure during autumn bears a considerable risk for colony failure as well, because virgin queens may not be sufficiently mated during their mating flights due to a comparatively lower number of available drones. Queen replacement in the neonicotinoid treatment group in the presence of original queens, and without swarming, was also observed during spring 2012. This indication of impaired performance of previously neonicotinoid-exposed queens, together with a likely reduced mating success of replacement queens from the previous autumn, is thus considered to be responsible for the overall decelerated colony growth of the neonicotinoid treatment group one year after exposure. The causal patterns of queen failure could be manifold, including compromised immunity that has been shown to result in increased pathogen loads in honeybee workers [36], [68], [69], [72]. Taking into account that honeybee colony fate essentially depends on the queen, the long-term impact of neonicotinoids on queens we observed, resulting in 60% supersedure within one year, represents a novel finding of major importance that clearly deserves further research. Increased queen supersedure rates were shown upon sublethal pyrethroid exposure [116] and detrimental effects on queen development and performance have also been reported from exposure to commonly encountered in-hive acaricides [117], [118], [119], [120], some of which are known to have similarly negative influence like neonicotinoids on worker bees [47], [52], [108], [121]. General effects of neurotoxic insecticides on queens may thus not be fundamentally surprising. Queen failure has repeatedly been indicated to be an important aspect shaping the present enigma of widespread colony losses [22], [25], [122], which may also be influenced by neonicotinoids. While direct contact exposure to agrochemicals can be generally considered much more relaxed for queens compared to workers, this might be different regarding oral exposure to systemic compounds, such as neonicotinoids. Here, ingestion of contaminated food represents a principal risk for all developmental stages and different castes of honeybees. Moreover, the queen’s life span as well as her level of trophallactic interactions far exceed that of workers [123], which may predestine her as a potential sink of sublethal exposure to systemic insecticides through pollen-based queen nutrition. Our results suggest that sublethal neonicotinoid effects on queens can jeopardize colony fate in the long-term, and should thus not be ignored.

Compared to the need for maintaining productive queens within colonies, swarming is less relevant for practical beekeeping, especially commercial operations. Nevertheless, apart from drone mating success, colony splitting through swarming represents a true fitness estimate in honeybees. According to overall reduced colony strength, colonies that were exposed to thiamethoxam and clothianidin in the previous season exhibited a significantly lower swarming success.

### The role of honeybee genetics

The detection of significant interactions of the honeybees’ genetic background with the effects of chronic neonicotinoid exposure on both colony performance in the short-term and queen performance in the long-term was a major insight of our study. Honeybee strain B, *A. m. mellifera* sister queens originating from a Swiss alpine region, was much more susceptible to the combination of thiamethoxam and clothianidin compared to strain A, *A. m. carnica* sister queens originating from a German region characterized by intense agriculture. For example, neonicotinoid-exposed colonies of strain B had the lowest numbers of adult bees directly after exposure (Fig. 1A), and none of their queens survived throughout the one year post-exposure period. This indicates that honeybee susceptibility to the here applied neonicotinoids may also include a genetic or epigenetic component, as has been previously suggested for neonicotinoids and other pesticides [17], [93], [111], [124], [125], [126]. It remains unresolved to what extent the outcome of our study was influenced by the usage of different honeybee ecotypes and/or by their distinct breeding histories linked to different environments. It could be possible that *A. m. mellifera* tends to be more susceptible to oral neonicotinoid exposure than *A. m. carnica* bees, as similarly found in another recent study [126]. Yet, compared to strain B, the particular breeding regime of strain A prior to our study may have simply included an unintended selection for higher neonicotinoid tolerance as a non-lineage-specific trait. Further research is required to explore the potential genetic basis underlying variable responses to sublethal neonicotinoid exposure in honeybees. Causal patterns could include detoxification genes and corresponding expression profiles, e.g., cytochrome P450 monooxygenases [93], yet, compared to many other insects, honeybees are equipped with a limited set of detoxification genes [127] and specifically the pathways for nitroguanidine neonicotinoid metabolism in honeybees remain less resolved compared to other insecticidal compounds [61], [111], [128], [129], [130], [131].

### Conclusions

In line with a recent meta-analysis [44], our results clearly indicate that neonicotinoids negatively impact on honeybee colony performance after chronic sublethal exposure throughout two brood cycles. Virtually all tested contrasts (18 out of 21) produced negative estimates for the effects of exposure to thiamethoxam and clothianidin (Table 1). It is supposed that sublethal neonicotinoid exposure through pollen has a stronger impact at the honeybee colony level compared to nectar-substitute feeding (e.g., [132]). Therefore more studies focussing on effects of sublethal exposure of larvae or nurse bees extending to the performance of complex tasks of adult forager honeybees are needed. Similarly, sublethal effects of neonicotinoids on honeybee queens clearly deserve in depth investigation.

It remains uncertain whether the observed colony level responses were stronger influenced by either thiamethoxam or clothianidin, or by the possibility for interactive effects of the combination of these neonicotinoids, both being ranked as having high risk potential to honeybees [64]. Yet, since clothianidin is the major metabolite of thiamethoxam, both bioactive compounds will be present in pollen and nectar of crops treated with thiamethoxam [80], [82], [96], [97], [98]. Thus, the combined exposure as well as the residue levels administered in pollen patties in this study represent a biologically relevant exposure scenario.

Exposure to almost any sublethal dosage of these highly potent insecticides could trigger adverse chronic effects in a time-dependant context [133], [134]. Our experimental exposure to contaminated pollen over 1.5 months can be considered as a worst-case scenario for agricultural settings, where honeybee colonies may encounter neonicotinoid-treated crops repeatedly throughout the season, but likely for shorter individual exposure periods. In bumblebees, it has recently been shown, that microcolonies can recover after short periods of sublethal exposure [135] to imidacloprid, which may well be similar, or even more pronounced in honeybee colonies. In a similar way, the supposedly exclusive exposure to pollen contaminated with neonicotinoids in our setup can be regarded as a worst case because in the field honeybee colonies generally exploit multiple available pollen resources at any time, some of which may only occasionally be contaminated with agricultural pesticides [38]. Nonetheless, it remains unclear whether repeated pulsed exposures over the entire season, either through consecutively available neonicotinoid-treated honeybee-attractive crops providing pollen and nectar (e.g., oilseed rape, sunflowers and maize that may each flower for 2–3 weeks) or through the general accumulation of neonicotinoid residues in the environment [38], [41], [42], [82], could result in detrimental effects as found here after 1.5 months of continuous chronic exposure. Moreover, although distinguishing between continuous and shorter but repeated periods of neonicotinoid exposure could represent different risk potentials for the honeybee worker population succumbed to generally high turn-over rates [101], the suspected buffering capacities at the colony level may deplete in the long-term, if the queen was still affected through pulsed exposures. Thus, a worst case scenario that applies realistic exposure levels while settling at the upper possible range of overall exposure duration deserves consideration. Currently there is no evidence that field-realistic neonicotinoid exposure could be directly involved in colony losses [79], [80], [81], [82], [83]. However, available studies do not fully allow to address the question of whether neonicotinoids are really contributing to colony weakening, because several aspects in corresponding experimental designs remain unsettled, such as lacking sufficient statistical power [44], the putative influence of honeybee genetics (see above), too small distances between test fields and other factors that challenge the prerequisite of adequate control fields, such as no pesticide treatment at all [41], [79], [80], [81], [82], queen effects that remain elusive [25] or queen rotation practices that impede uncovering potential effects on queen performance [80]. Interestingly, this long-term impact detected in our study, notably supersedure of failing queens, compounds recent criticism that the presence of a given predictor may not necessarily correlate with impaired colony function [65] by showing that delayed effects can emerge also in the absence of causal stressors, in this case the lack of traceable amounts of the applied neonicotinoids within hives several weeks after exposure. There is an urgent need for more thoroughly designed studies to clarify the threats of neonicotinoids to honeybees, and pollinators in general [42]. The growing body of scientific awareness on sublethal side-effects of pesticides on non-target pollinators, ranging from gene expression profiles of individual developmental stages to entire life-time fitness performances of different species of bees, represents a unique opportunity to benefit the current framework of pesticide risk assessment [64], [84], [136], [137].

Finally, the here detected interactions of honeybee genetics and impacts of chronic neonicotinoid exposure on colony performance suggest that there is genetic variability for neonicotinoid susceptibility, and thus potential to partly counteract negative effects through selective breeding of more tolerant honeybee strains.

## Supporting Information

Figure S1 **Colony set up at the experimental apiary.** Two groups of honeybee colonies each comprising of 7 colonies originating from strain A (blue squares) and 5 colonies originating from strain B (yellow squares) were placed in a row in front of a small forest with entrances pointing in the same direction. All colonies were maintained approximately one year before and one year after the experimental feeding with either control or neonicotinoid-spiked pollen patties over two brood cycles.
(EPS)
